# Misinformation in the media: global coverage of GMOs 2019-2021

**DOI:** 10.1080/21645698.2022.2140568

**Published:** 2022-11-17

**Authors:** Mark Lynas, Jordan Adams, Joan Conrow

**Affiliations:** a The Alliance for Science, The Boyce Thompson Institute, Ithaca, New York, USA; b Cision Global Insights, Ann Arbor, Michigan, USA

**Keywords:** Biotechnology, genetic engineering, genetic modification, GMO, media analysis, media coverage, misinformation, sentiment analysis, agricultural, biotechnology

## Abstract

Misinformation is a serious problem in scientific debates ranging from climate change to vaccines to nuclear power. In this study we quantitatively assess the phenomenon of misinformation – defined as information which is at variance with widely-accepted scientific consensus – on genetically modified crops and food (“GMOs”) in the mainstream and online news media over a two-year period. We found an overall falsehood rate of 9% with a potential readership of 256 million. None of the misinformation was positive in sentiment; most was negative. About a fifth of Africa’s media coverage on GMOs contained misinformation, a worrying finding given the potential for genetic engineering to deliver improved nutrition and food security in the continent. We conclude that misinformation about GMOs in the mainstream media is still a significant problem, and outranks the proportion of misinformation in other comparable debates such as COVID-19 and vaccines.

## Introduction

Misinformation can damage society’s interests because people who are misinformed about an issue may make decisions based on flawed or false information.^1^ For example, the World Health Organization declared an “infodemic” during the initial stages of the COVID-19 pandemic due to the proliferation of misinformation about the disease, its causes, and possible treatments.^2^ This “infodemic” led to increased death rates and disease due to people seeking and applying inappropriate treatments and ignoring and resisting evidence-based control measures.^3^

Misinformation does not necessarily arise primarily from the grassroots; powerful people and interests can play an influential role in originating and spreading it. Our earlier work on COVID-19 found that the greatest single purveyor of misinformation in the year up to October 2020 was the then-president of the United States, Donald Trump, who infamously promoted bleach and hydroxychloroquine as a treatment for the disease, among other misinformation.^4^

COVID-19 vaccines have also been the subject of extensive misinformation, despite having saved an estimated 20 million lives within a year of deployment.^5^ Misinformation about vaccines is undoubtedly a killer; some of those who avoided getting safe and effective COVID-19 vaccines died from the disease unnecessarily. Analysis has found at least a quarter of a million vaccine-preventable deaths have taken place during the pandemic in the United States alone.^6^

On a different issue, misinformation about climate change has undermined the political consensus needed to mitigate the damage caused by global heating. While 99.9% of the peer-reviewed literature no longer disputes that humans are the primary cause of climate warming,^7^ polls show the general public is unaware of this high degree of consensus,^8^ with denialism being particularly strong where political ideology comes into play.^9^

The vast majority of scientific research on the phenomenon of misinformation focuses on social media, which is acknowledged to be less reliable than mainstream media because peer-to-peer content is mostly not subject to gatekeeping and fact-checking by professional editors. A large volume of work has been published on COVID-19 misinformation and vaccine misinformation, all looking at social media.

Fewer researchers have examined the phenomenon of misinformation in traditional mainstream and online news media, perhaps due to the assumption that this is not a significant problem. Our earlier work on vaccines suggested that only about 0.1% of COVID-19 vaccine articles in traditional and online media might contain “primary misinformation” (i.e., unchallenged misinformation). This did not mean the issue was unimportant, however, since even a small number of articles can reach millions of people. We concluded that “COVID-19 vaccine misinformation in the traditional news media is uncommon but has the capacity to reach large numbers of readers and affect the vaccine conversation.”^10^

Genetically modified crops have been subject to a decades-long, orchestrated campaign of misinformation by opponents. This has yielded substantially negative public attitudes and media coverage and resulted in biotech regulatory systems that are de-facto prohibitionary in many geographies from Europe to sub-Saharan Africa.^11,12^ While there is some evidence that the media conversation on GMOs has become less salient and less polarized in recent years,^13^ alongside evidence of declining concern among consumers,^14^ there is still a substantial amount of opposition that may be driven or exacerbated by media-originated misinformation.

Expert consensus can help define misinformation where there are competing assertions of fact in areas of science. For example, if 99.9% of literature on climate change does not dispute the human cause,^7^ this tells us that those who do evoke alternative causes or deny that the climate is changing at all are far outside the scientific mainstream. Scientific consensus can be built and conveyed by international expert groupings like the Intergovernmental Panel on Climate Change^15^ or the national academies of science of different countries. It can also be deduced from meta-analyses and well-constructed literature reviews that bring together the results of multiple scientific studies to draw an overall conclusion.

An overview published in 2015 of a decade of GM safety studies found no evidence of significant hazards connected to the use of engineered crops.^16^ Meta-analysis of the ecosystem impacts of growing GM corn/maize has shown no discernible negative impact on non-target invertebrates and beneficial insects, particularly when compared to conventional treatments utilizing chemical insecticides.^17^ Likewise, there is a clearly stated consensus among major national and international scientific bodies that food derived from GM crops is as safe as any other. This is expressed variously in statements and assessments by the US National Academy of Sciences,^18^ the AAAS board,^19^ the World Health Organization,^20^ and the UK Royal Society,^21^ among many others. Thus, claims that GM crops and foods are harmful to health, for example, can properly be termed misinformation when lacking widely accepted supporting scientific evidence.

Some studies have been published over the years that do purport to show health risks from GM crops. The most famous of these (the so-called Seralini study^22^) was retracted due to numerous methodological flaws, but those that remain in the scientific record have been shown to be without statistical evidence.^23^ Other papers purporting to show harms have also been retracted, published in predatory journals with minimal or poor peer review, or found to contain plagiarism or even outright fraud.^24^ As an extensive 2017 review of those scientific studies which are often cited by opponents as evidence of adverse effects of GM foods concludes, “a close examination of these reports invariably shows methodological flaws that invalidate any conclusions of adverse effects.”^25^

There have been no recent publications to our knowledge that would alter such an assessment. Thus, there is no scientific basis to claims of harm associated with the production and/or consumption of so-called GMOs. In our view, such claims can properly be termed “misinformation” when appearing in the popular media without qualification or rebuttal.

This study aims to quantify the extent of this misinformation in a selection of top worldwide, online English language news media. We use the term “misinformation” in a broad definition to include both “mis” and “dis” information (the latter is usually termed to include intentional falsehood, while the former may be unintentional), and use the term “misinformation” to label content in news media that is at variance with generally-accepted scientific facts on the GMO issue, as discussed above.

## Methods

To build up a database of articles, we searched the Cision Media NextGen platform to identify every article from a pre-determined list (see Supplementary Information for the full list) of top English-language media that contained three or more GMO-related keywords. The attributes of the NextGen platform are described in previous papers by some of the same authors, in particular Lurie, et al., 2022^10^ and Evanega, et al., 2022.^13^ We added a selection of top news media from anglophone sub-Saharan African countries to more closely evaluate GMO misinformation in this region.

A Boolean search string on GMO topics was developed and iteratively refined to sift out irrelevant returns and build up a database. (See Supplementary Information for the full Boolean string.) We used a 2-year period from January 12, 2019 to January 12, 2021, which we felt was recent enough to be of current interest and long enough to support meaningful conclusions, while not returning so many articles as to make detailed manual analysis too laborious.

Our search string returned 535 relevant articles, which were then categorized into 5 topics, namely “human health,” “environment,” “production,” “pesticides,” “animal health,” and “miscellaneous,” according to their content. Articles fitting into more than one category were included more than once. Categories are described below:

- “Human health” included articles focusing on genetically modified crops and food as they relate to human health, particularly regarding the consumption of GM-originated foodstuffs. These might cover claims and counterclaims in areas such as cancer, nutrition, and obesity, or benefits to health such as biofortification or a reduction in aflatoxins.
- “Environment” included articles focusing on how the cultivation of GM crops impacts the environment, encompassing such issues as cross-pollination and impacts on soils and wildlife.
- “Production” included articles focusing on genetically modified crops as they relate to crop yields, food security, control of seeds, and global food supplies.
- “Pesticides” included articles covering the relationship between GM crops and pesticides, such as herbicide tolerance and insect-resistance traits, and issues arising from glyphosate specifically regarding its use on GMOs.
- “Animal health” included articles discussing GMOs as they relate to animal health, particularly the consumption of GM feeds by animals. These included animal feeding trials and GMOs in feed generally.
- “Miscellaneous” included GMO mentions not included in the topics above, such as financial discussions of companies, product recalls and GMO product approvals and regulation.

Articles were manually assigned a category and also manually coded for sentiment, with tags of “positive,” “negative,” “neutral,” or “mixed” being assigned. In our definition, “positive” would likely sway an undecided reader to support genetic engineering in food and crops, while “negative” would likely sway a reader to oppose it. (These are likely to include opinion pieces and “good” or “bad” news stories about the content.) “Neutral,” a sentiment characteristic of the majority of news reportage, would not be expected to sway a reader either way, while “mixed” would include opposing viewpoints from either side.

We also recorded the date, region/country, media outlet, title, URL, and article type of each story. The latter primarily included news and opinion, with a small number of sponsored pieces and interviews also appearing in our database.

We present the results below both in terms of volume of articles and readership. Volume is the absolute number of articles published, although an article may be counted twice or more if it falls into more than one issue category (e.g., if it discusses both human health and environment themes it will appear in both those categories). We define readership as the total potential reach of a media item, whether print or online. For a full description for how readership is calculated for this approach, see “gross reach” in Evanega. et al., 2022.^13^

## Results

We found a total of 535 relevant articles for the 2-year period in which we searched our media database with the GMO Boolean search string. Of these, we rated 488 as factual and 47 as containing misinformation (see Supplementary Information for a spreadsheet listing all articles and their ratings/categorizations). Overall, about 9% of the articles published on GMOs from 2019–2021 that we reviewed contained misinformation, while 91% were factually accurate. In terms of readership, the articles we rated as factually accurate had a potential readership of 4.8 billion, while those containing misinformation had a reach of 256 million. In percentage terms, misinformation was therefore about 5% of our total readership (Table 1).

**Table 1.** Occurrence of GMO misinformation, January 2019 – January 2021.

	Factual	Misinformation	Total
Articles number	488	47	535
Percentage	91%	9%	100%
Readership volume	4.8 billion	256 million	5 billion
Readership percentage	95%	5%	100%

**Figure 1.**
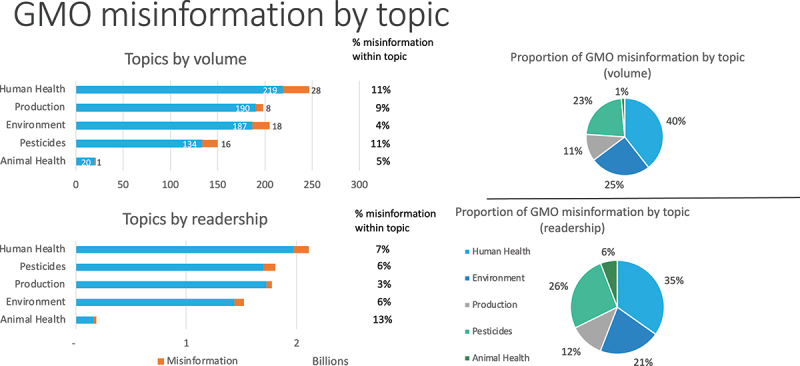
GMO misinformation totals by topic.

Most instances of misinformation returned in our search fell into the category of “human health,” with 28 instances of misinformation out of a total 247 articles covering this issue. This means that 11% of our database of media coverage of GMOs mentioning human health was tagged as misinformation. A similar proportion of the “pesticides” category (also 11%) was tagged as misinformation, although in volume terms the number (16 articles out of 150) was lower. Note in Fig. 1, which shows the volume and readership by topic of GMO misinformation, that the total number of GMO topic mentions in volume terms (821) is greater than our total number of articles (535) because some articles reference more than one topic and so are counted more than once.

Figure 1 also shows the proportional occurrence of the different topics within the overall “misinformation conversation.” Thus 40% of the “misinformation conversation” on GMOs in traditional media fell into the “human health” topic category and 25% into the “environment” category in terms of volume. In readership terms, 35% of the misinformation was about human health, 26% about pesticides, and 21% about environment.

Misinformation on GMOs and human health also had the highest readership, with 139 million out of 2.1 billion potential views. In quantity terms, misinformation about pesticides had the next highest readership, with 106 million out of 1.8 billion potential views. Misinformation on environment achieved 85 million out of 1.5 billion readership, while misinformation on production had 47 million out of 1.8 billion views and misinformation on animal health 23 million out of 185 million readership. In terms of the percentage of misinformation within the topic, animal health ranked highest by readership at 13%, although this is based on a low volume of articles (1 misinformation out of 21 total articles), with human health second on 7% and pesticides and environment both on 6%.

**Figure 2.**
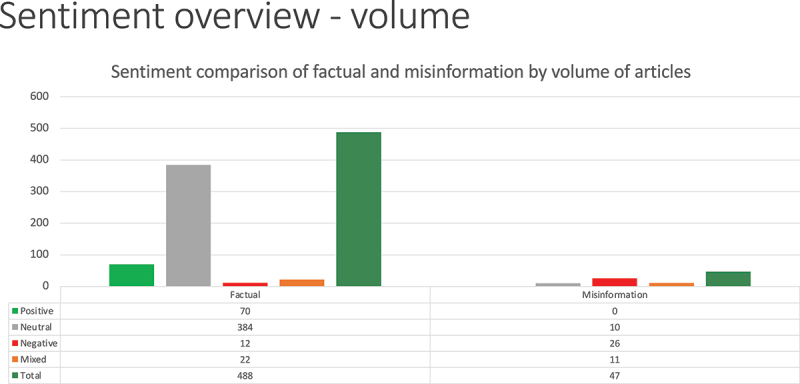
Sentiment of GMO articles by volume.

We also analyzed the sentiment of the content we reviewed. As shown in Fig. 2, the overwhelming majority of factual articles were rated as “neutral,” likely encompassing the media’s straightforward approach to reporting on the GMO issue. In the misinformation category, the majority of articles were characterized as “negative.” It is notable that no misinformation was rated as “positive.” In other words, we found that all misinformation about GMOs in traditional media had a negative, mixed, or neutral tone.

**Figure 3.**
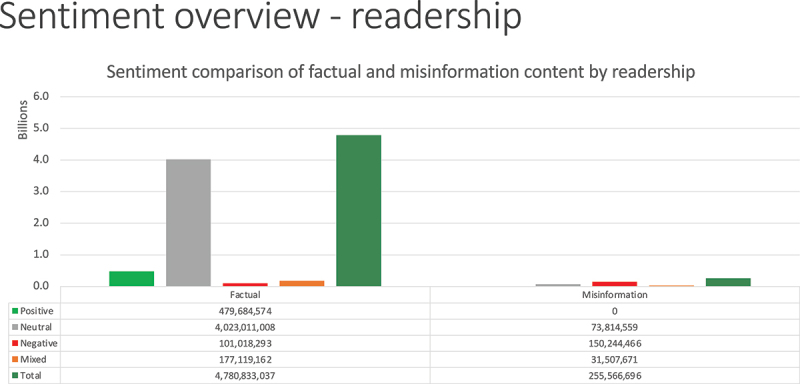
Sentiment of GMO articles by readership.

Sentiment by readership is illustrated in Fig. 3, which shows that factually accurate neutral news coverage accounted for the majority – about 4 billion – of reader views. While combined negative and mixed sentiment misinformation achieved a readership of 181 million, this was still outweighed by positive factual readership of 480 million. Note that not all negative coverage was rated as misinformation. There was a potential readership of 101 million for factually accurate articles we rated as “negative” and an additional 177 million for articles rated as “mixed.”

**Figure 4.**
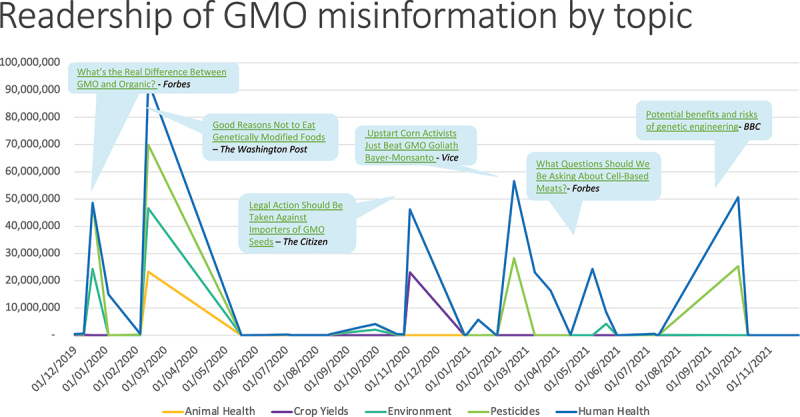
Topic spikes and readership for the highest visibility misinformation articles.

In Fig. 4 we show some of the topic spikes in misinformation content by readership. Spikes corresponding to the publication of the highest-visibility misinformation articles are labeled on the chart, and the lines are color-coded by topic. Note that articles may contain more than one topic, so totals are again higher than the combined readership for all misinformation content due to double counting. (Note that this does not affect the totals in Figs. 2 and 3, which are not broken down by issue topic and therefore no articles are counted more than once.)

**Figure 5.**
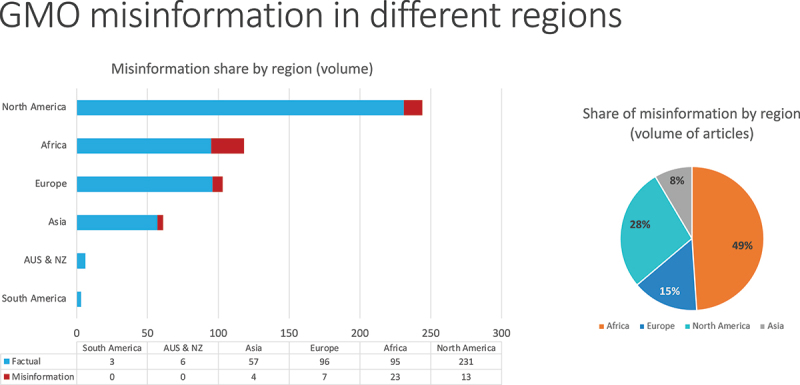
GMO misinformation by volume, broken down by region.

We also looked at the volume of articles broken down by region (Fig. 5). While the majority of articles returned in our search of English-language media were produced in North America, it is striking that Africa produced the highest proportion of misinformation in its coverage. About 20% – a fifth – of Africa’s GMO media content in our database is rated as misinformation. The corresponding proportion is 5% for North America and 7% for Europe.

While it may appear striking that nearly half of all misinformation surveyed arose in Africa (see pie chart on Fig. 5), we cannot make a true comparison to other regions as we intentionally highlighted African media for special investigation in our media list, thus biasing it for this purpose. This should be borne in mind when interpreting Fig. 5, which shows the volume of misinformation in different regions and contains a higher proportion of articles from Africa that would likely be returned in a straightforward global search of top online news media.

## Discussion

Our analysis found a 9% occurrence of misinformation in volume terms in a two-year period of media analysis, meaning that 91% of the coverage we reviewed was deemed factually accurate. This is a substantially higher proportion of misinformation than has been found in other scientific debates. As noted earlier, our work on vaccine misinformation found a rate of occurrence of only around 0.1% in the mainstream media,^10^ although methodological differences mean these figures are not like for like comparable.

For climate change, recent studies investigating misinformation/climate denialism in the media have found rates of skepticism about anthropogenic global warming within a similar range to our results.^26^ Our earlier study on the COVID-19 “infodemic” found that 2.9% of the media coverage of the pandemic related to misinformation, though this figure included fact-checking and articles about the “infodemic” itself.^4^ This suggests that the phenomenon of GMO misinformation has been substantially worse than the COVID-19 “infodemic” in terms of the proportion of false information disseminated by the media.

One of the most striking results from our analysis is that 100% the misinformation about GMOs has been characterized as negative, mixed, or neutral in sentiment, while none has been positive. This suggests that anti-GMO activist networks have been successful in persistently influencing media coverage of the issue, and that misinformation primarily originates from the anti-GMO perspective.

Even relatively low levels of misinformation about genetic engineering are a concern because the resulting avoidance of the technology in agriculture has had negative real-world impacts that have been evaluated and quantified. A meta-analysis of the global impact of GM crops found an average yield increase of 22% and a reduction in chemical pesticides of 37%.^27^ These benefits are foregone in places where so-called GMOs are banned. With all other things remaining equal, the avoidance of agricultural biotechnology will therefore result in more chemical use and lower yields than would be the case with full adoption.

It has been calculated, for instance, that Europe’s non-adoption of GM crops led to the emission of 33 million more tonnes of CO_2_-equivalent greenhouse gases than would otherwise be the case.^28^ A recent global assessment of GM crops found that they have reduced pesticide spraying by 775.4 million kg (8.3%) and reduced greenhouse gas emissions equivalent to removing 15.3 million cars from the roads for one year.^29^ Net economic benefits of GE crops at the farm level totaled an estimated $18.9 billion in 2018, equally divided between developed and developing countries.^30^

Developing countries that have adopted GM crops show clear benefits to the technology. The adoption of Bt brinjal in Bangladesh has dramatically reduced pesticide sprays and led to a six-fold increase in the profits of the smallholder farmers who grow the crop.^31^ The adoption of Bt cotton in India, despite being subject to a campaign of misinformation by anti-GMO activists blaming it for a supposed increase in farmer suicides,^32^ has in fact increased farm incomes and helped avoid several million cases of pesticide poisoning every year in India.^33^ Reductions in farmer pesticide poisonings attributable to the adoption of GM crops also have been quantified in China, Pakistan, and South Africa.^34^

Our results are especially concerning in that they show the persistence and influence of anti-GMO misinformation in sub-Saharan Africa, quantitatively confirming anecdotal observations made by practitioners in the field. Roughly a fifth of the media coverage in Africa contained false messages about GMOs, confirming the influence of anti-GMO activism in the continent and at least partially explaining the negative policy environment applied to GM crops and food in most African countries. This finding raises ethical questions given the benefits that genetically engineered crops have delivered to smallholder farmers in other parts of the world and the fact that many anti-GMO groups in Africa are supported by groups based in the Global North, primarily Europe, where food security is not an issue.^35^

Many of the African misinformation articles we found quote extensively from campaigners who are part of these NGO networks based in the Global North. Current campaigns against GM cowpea would, if successful, reduce protein intake and hamper the ability to tackle malnutrition and health and ecological impacts arising from the overuse of pesticides.^36^ GMO benefits foregone could be particularly substantial in sub-Saharan Africa; one study has found that a delay in the approval of GM cowpea could cost the country several thousand lives.^37^ This problem is not unique to GMOs. Misinformation about vaccines also drives vaccine hesitancy in sub-Saharan Africa.^38^

Much of the anti-GMO coverage in the media is based on skeptical statements by self-proclaimed experts denying the existence of scientific consensus on GMO safety (such as the so-called ENSSER statement.^39^) To give a fig leaf of scientific probity is presumably the motivation behind such statements, which have also been produced by climate skeptics^40^ as well as vaccine opponents. Both groups have curated long lists of selectively quoted peer-reviewed science to back up their ideological positions.^41^ Stated consensus is never 100% (although inferred climate change consensus comes tantalizingly close at 99.9%^7^) because individuals hold differing opinions: surveys of physicians have found 11% did not recommend vaccines for children,^42^ despite overwhelming evidence that vaccines are safe and effective.

Thus, there will always be bona fide experts available to be groomed as effective media performers who can give the impression that “the experts disagree” on key science issues. This is termed “false balance” and it represents a trap the media often fall into when they inadvertently misinform the public by reporting two apparently equal sides of an expert argument.^43^ Skeptics have thus become adept at using scientific-sounding terms or fake experts to spread misinformation. For example, a study on COVID-19 vaccine intent found that messages around “scientists expressing doubts” was particularly effective in undermining trust in vaccines.^44^

## Conclusion

Our analysis finds that GMO misinformation is still a significant problem in the media, with nearly a tenth of all coverage of agricultural biotechnology containing falsehoods at variance with widely accepted scientific facts. This rate of misinformation is far higher than that for vaccines and comparable to the highly politicized issue of climate change. Given that the potential worldwide readership of this misinformation totaled over a quarter of a billion between 2019 and 2021, it is incumbent on the scientific community to make urgent efforts to improve its communications about genetic engineering with both the media and the general public.

If misinformation is allowed to proliferate, resulting policy measures like GMO bans and other restrictive laws will undermine efforts to advance agricultural sustainability and food security worldwide by preventing practitioners from using this valuable technology. This is particularly concerning in Africa, where our analysis found misinformation rates as high as a 20%. With food security in sub-Saharan Africa still a major challenge, we suggest that scientific communications efforts should be particularly focused on the continent to decrease the rate of falsehoods about GMOs in African media coverage and thus improve the accuracy of information reaching policymakers and citizens.

## Supplementary Material

Supplemental Material

Supplemental Material
