# Supplementary material for: Misinformation in the media: global coverage of GMOs 2019-2021
Source: GM Crops Food. 2022 Nov 17;16(1):18–27. doi: 10.1080/21645698.2022.2140568 (PMC11702960; doi:10.1080/21645698.2022.2140568)
Supplement: Supplemental Material [file KGMC_A_2140568_SM1480.docx]

GMO misinformation study - Supplementary Information

Outlets surveyed

The following news media outlets were used in the initial search to source content. Outlets **not in bold** were included in our media list but did not return any results when searched with the search string below.

**ABC News**

Addis Fortune

**AJC**

**All Africa**

**ANI News**

Arusha Times

Asian Age

**Associated Press**

**BBC**

BD Live

**Bloomberg**

Boston Herald

**Business Daily Africa**

**Business Daily Kenya**

**Business Insider**

**Buzzfeed**

**Capital Ethiopia**

CBS News

**Chicago Sun Times**

**Chicago Tribune**

**City Press 24**

**CNBC**

**CNET**

**CNN**

CS Monitor

Daily Beast

**Daily Mail**

Daily Maverick

Daily News Tanzania

Daily Pioneer

**Daily-Mail Zimbabwe**

Dallas News

Deccan Chronicle

**Deccan Herald**

Denver Post

Devex

DNA India

Economist

**ENCA**

**Entrepreneur**

Ethiopia Press

**Euractiv**

**Express**

Fast Company

**Financial Express**

**Financial Times**

First Post

**Forbes**

**Foreign Affairs**

Foreign Policy

**Fortune**

**Fox News**

Frontline India

Ghana News Agency

**Grist**

**Guardian Nigeria**

**Hindustan Times**

**Houston Chronicle**

Huffington Post

**Humanosphere**

**IANS**

**IBTimes**

**Independent Nigeria**

**Independent UK**

India Today

India Today News

**Indian Express**

**IOL News**

**IPP Media**

LA Times

**Leadership Nigeria**

Mail Today India

**Mashable**

Mercury News

MG Africa

MG Zimbabwe

**Miami Herald**

Mirror UL

**Monitor Uganda**

MSNBC

**Mumbai Mirror**

My AJC

My Digital LFC

**Nation Kenya**

National Journal

NBC News

**NDTV**

New Indian Express

New Jersey Star Ledger

New Statesman

New York Daily News

New York Post

New Yorker

**Newsweek**

NPR

OC Register

**Outlook India**

**Politico**

Post Zambia

Propublica

**Punch Nigeria**

**Quartz**

**Reuters (All)**

SABC Zimbabwe

**Salon**

**Science Daily**

**Science Dev**

**Science Magazine**

**Seattle Times**

**SF Chronicle**

**Slate**

Spectator Ghana

Spectator UK

Standard UK

**Star FM Online**

Tamba Bay Post

Tehlka

**Telegraph UK**

The Atlantic

The Chronicle (Ghana)

**The Citizen**

**The Guardian**

**The Hill**

**The Hindu**

The Lancet

**The Nation**

**The New York Times**

**The Star Kenya**

**The Statesman**

**The Sun**

The Times UK

**This Day Live**

**Time**

**Times of India**

**US News**

**USA Today**

**Vanguard Nigeria**

Ventures Africa

**Vice**

**Vox**

Wall Street Journal

**Washington Post**

Washington Times

**Wired**

**Yahoo (All)**

**Zambian Eye**

News outlets that published misinformation according to our search

(for specific article hits from these sources, including misinformation flags and URLs see Supplementary Info spreadsheet)

- Ani News
- Grist
- Tanzania Citizen
- VICE
- Salon
- Outlook India
- Capital Ethiopia
- Yahoo Canada
- Vanguard
- Slate
- This Day Live
- Punch Nigeria
- Buzzfeed
- IPP Media
- Financial Times
- The Washington Post
- The Guardian
- BBC News
- Euractiv
- Bloomberg
- The Star
- Times of India
- AllAfrica
- Leadership Nigeria
- Forbes
- Yahoo News

Boolean search string

**GMO Keyword Search:** (keyword:(("frankenfood" OR "frankenfoods" OR "GMO" OR "GMOs" OR "Genetically modified organism" OR "genetically modified organisms" OR "genetic engineering" OR "genetically engineered" OR "genetically modified" OR "genetic modification" OR "transgenic" OR "agricultural biotechnology") **NOT** ("sgRNA" OR "coronavirus" OR "covid" OR "mosquitoes" OR "mosquito" OR "mice" OR "mouse" OR "non gmo"~1 OR "GMO Internet" OR "pandemic" OR "lungs" OR "Anopheles" OR "weapons" OR "SARS-CoV-2" OR "autoimmune" OR "rodent" OR "grantham" OR "grantham's" OR "Oregon" OR "aging" OR "opossums" OR "bear" OR "bears" OR "squids" OR "squid" OR "aldous" OR "Elon" OR "poplar" OR "zombies" OR "glioblastoma" OR "bitcoin" OR "psybio" OR "astronomer" OR "Philip Morris" OR "phytohormone" OR "spidroins" OR "cytokines" OR "salicylic" OR "endothelial" OR "blockade" OR "zebrafish" OR "vivo" OR "polypeptides" OR "ions" OR "gamma" OR "peptides" OR "biotrophic" OR "polarized" OR "monkey" OR "pluripotent" OR "stemness" OR "squirrels" OR "zoetis" OR "silkworm" OR "ester" OR "printing" OR "isolation" OR "phenotypic"))
